# Clinical prediction for outcomes of patients with acute-on-chronic liver failure associated with HBV infection: A new model establishment

**DOI:** 10.1515/med-2020-0207

**Published:** 2020-07-20

**Authors:** Wenhan Fan, Wei Liao, Yiping Luo, Benming You, Jiao Yu, Chengzhong Li

**Affiliations:** Department of Infectious Diseases, The First Affiliated Changhai Hospital of The Second Military Medical University, Shanghai, China; School of Basic Medical Sciences, The Second Military Medical University, Shanghai, China; Department of Medicinal Materials, The First Affiliated Changhai Hospital of The Second Military Medical University, Shanghai 200433, China

**Keywords:** hepatitis B virus infection, acute-on-chronic liver failure, risk prediction

## Abstract

**Objective:**

The acute-on-chronic liver failure associated with hepatitis B virus (HBV-ACLF) was a type of clinical syndrome with rapid deterioration of liver function. It was characterized by short-term elevated bilirubin, ascites, prolonged clotting time, hepatic encephalopathy, organ failures, and high short-term mortality. It was important to predict and evaluate the disease early. This study intended to comprehensively analyze the prognostic factors of patients with ACLF associated with HBV DNA infection through clinical manifestations and laboratory tests, and to establish a corresponding prediction and evaluation model for further clinical guidance.

**Methods:**

A total of 220 patients were first diagnosed with HBV-ACLF and admitted to and treated at the Department of Infectious Diseases of the First Affiliated Changhai Hospital of the Second Military Medical University from 2009 to 2018. These patients’ records were collected and divided into two groups: (1) 120 patients who were improved and discharged were classified as good prognosis group and (2) 100 patients who died or underwent liver transplantation were classified as poor prognosis group. By analyzing baseline characteristics and clinical indicators of the two groups, the main potential factors affecting prognosis were identified and the corresponding prognostic evaluation model was established. This model’s advantages and disadvantages were compared with classic prognostic scoring systems.

**Results:**

The proportion of ascites and the proportion of hepatic encephalopathy of poor prognosis group were significantly higher than those of good prognosis group. The total bilirubin, creatinine, white blood cell count, and NEU (%) levels of poor prognosis group were significantly higher than those of good prognosis group, and the international normalized ratio, albumin (ALB), alanine aminotransferase, Na, Cl, RBC, and PLT levels of poor prognosis group were significantly lower than those of good prognosis group. A new prediction model LR(*p*) = 1/(1 + *e* ^−*Z*^) was established, where *z* = 10.0127 + 0.3687 × NEUT (%) − 0.0082 × PLT + 1.8157 × hepatic encephalopathy. The area under receiver operating characteristic (ROC) curve was 0.89, specificity was 80.83%, and sensitivity was 81%. The newly established prognostic model was compared with other three scoring systems including model for end-stage liver disease (MELD), MELD-Na, and ALBI scores. The results showed that the specificity, sensitivity, and area under the ROC curve of the newly established model were significantly higher than the other three scoring systems.

**Conclusion:**

Hepatic encephalopathy, NEU (%), and PLT levels were independent risk factors for predicting the prognosis of HBV-ACLF. The new prediction model LR(*p*) had better prediction accuracy than the other three scoring models of MELD, MELD-Na, and ALBI and could more accurately assess the prognosis of HBV-ACLF, but in the later stage, it was still necessary to expand the sample size for verification.

## 1 Introduction

Acute-on-chronic liver failure (ACLF) is a type of clinical syndrome based on the chronic liver disease with a rapid deterioration of acute liver function due to various factors [1]. It is clinically characterized by rapid onset, dangerous condition, and high mortality. In the past 10 years, with the wide application of antiviral drugs, systematic treatment of internal medicine, and increasingly mature artificial liver and liver transplantation, the survival rate of patients has been improved to some extent. However, its clinical short-term mortality is still high [2,3].

The morbidity of hepatitis B in the Asia-Pacific region is especially high; therefore, HBV infection becomes the most important cause of ACLF. ACLF progresses rapidly, and the mortality is as high as 50–90% [4]. If early timely diagnosis and treatment is not available, liver transplantation becomes the only effective treatment regimen for various complications and organ failure in the later stage. However, due to limited liver resources and high cost, most patients with ACLF still rely on internal medicine and artificial liver treatment. Artificial liver support treatment can temporarily replace a part of the liver function, improve the micro-environment of liver cell regeneration and liver function repair by eliminating toxic substances and metabolites in serum, and prevent liver failure from further deterioration. However, the efficacy of artificial liver support for liver failure treatment is still controversial [5,6,7]. Therefore, early diagnosis and assessment of the disease, understanding of the occurrence and development factors, and the establishment of accurate predictive models and evaluation systems can provide guidance for later clinical treatment.

Due to the complicated occurrence and progression mechanism of AFLC, the disease evolved rapidly, and the lesions could involve multiple organs and tissues. The assessment of a single indicator could not fully and accurately reflect the severity and prognosis of the disease. Therefore, it was necessary to further establish a corresponding multifactor prognosis model through the statistical method to improve the specificity and the sensitivity of the prediction. Currently, many classical models were used to evaluate ACLF, and some models were modified and improved based on classical models, but there were still some shortcomings and deficiencies [8,9].

The earliest Child-pugh (CTP) score had been used clinically for more than 50 years. The evaluation indicators included serum total bilirubin, albumin level, hepatic encephalopathy, ascites, and prothrombin time (PT). So far, it was still one of the classic models used by the internal medicine department and the surgery department to quantify and assess the liver reserve function of patients with cirrhosis. However, there were certain defects. For example, ascites could be affected by diuretics, and albumin level could be changed by intravenous infusion. In addition, the renal function was not reflected in the model, which caused the CTP score not to fully reflect the severity and prognosis of the disease. Model for end-stage liver disease (MELD) and its derived MELD score were originally used to predict the mortality within 3 months after surgery in patients undergoing transjugular intrahepatic portosystemic shunt, which were subsequently found to be used to determine the prognosis and prioritization of liver transplantation. Compared with the CTP score, MELD was more refined and more accurate in predicting the prognosis of end-stage liver disease. Hence, Biggins et al. [9] improved the MELD score and established the MELD-Na score by combining with serum Na, which could more accurately assess the prognosis of end-stage liver disease compared with MELD, but had limited application value for patients with early liver disease. In 2013, the MELD-Na score was improved by the EASL Liver Failure Group based on the sequential organ failure assessment score (SOFA), and they derived the assessment model chronic liver failure–sequential organ failure assessment score (CLIF-SOFA) for liver failure. In recent years, the hepatic encephalopathy, international normalized ratio, neutrophil–lymphocyte ratio, age, and total bilirubin, termed as the HINAT ACLF mode (HINAT-ACLF) model, established by Gao et al. [10] had shown strong predictive value in screening out five independent risk factors including hepatic encephalopathy, INR, neutrophil lymphocyte ratio, age, and TBil. In another study, Wu et al. [11] analyzed the clinical materials of 1,322 inpatients with acute decompensated cirrhosis or chronic hepatitis B (CHB) liver injury in 13 liver disease centers, developed a new prognostic model, and diagnosed nearly 20% of patients with hepatitis B with ACLF incentives, thereby increasing their chances of receiving timely intensive treatment.

This study retrospectively analyzed the acute-on-chronic liver failure associated with hepatitis B virus (HBV-ACLF) patients admitted to and treated at the Department of Infectious Diseases of the First Affiliated Changhai Hospital of The Second Military Medical University from 2009 to 2018. Through data mining the baseline information, laboratory indicators, and related factors affecting prognosis of HBV-ACLF patients with different prognosis, we established a predictive model, compared it with other models, and investigated its application value.

## 2 Subjects and methods

### 2.1 Patient selection

The medical records of inpatients with first diagnosis in line with the HBV-ACLF criteria at the Department of Infectious Diseases from 2009 to 2018 were retrieved in the clinical data record system of the First Affiliated Changhai Hospital of The Second Military Medical University. The main laboratory test data, changes in the condition and clinical medication during hospitalization, and conditions at discharge were also collected. This study was approved by the Ethics Committee of Changhai Hospital, and all patients signed the informed consent form.

### 2.2 Inclusion and exclusion criteria

Because the enrolled patients were Asian population, the inclusion and exclusion and diagnostic criteria of HBV-ACLF patients were based on the *Guidelines for the Prevention and Treatment of Chronic Hepatitis B* [12] issued by China in 2015 and the *Guidelines for the Diagnosis and Treatment of Liver Failure* [13] issued by China in 2012. The enrolled patients had a history of CHB infection, with HBsAg or HBV DNA positive for more than 6 months, and may have complication of underlying cirrhosis, with serum total bilirubin TBil ≥ 10 × ULN or daily increase of ≥17.1 μmol/L and PTA ≤ 40% (or INR ≥ 1.5). Patients with viral hepatitis, alcoholic liver disease, autoimmune liver disease, drug-induced liver damage, tumors, or severe organ diseases other than hepatitis B were excluded. Each patient must be hospitalized for at least 1 week.

### 2.3 Demographic information

Basic information included sex and age of patients.

### 2.4 Clinical indicators

At admission, patients were screened for hepatic encephalopathy, ascites, and cirrhosis. Liver cirrhosis and ascites were determined by CT/MRI/B ultrasound tests. Hepatic encephalopathy was determined through clinical inquiry about directional power and calculation power, combined with blood ammonia index. The first collected venous blood test results were recorded: including baseline white blood cell count (WBC), neutrophil ratio (NE%), lymphocyte ratio (LY%), eosinophil ratio % (EO%), alanine aminotransferase (ALT), aspartate aminotransferase (AST), γ-glutamyltranspeptidase (γ-GT), total bilirubin (TBil), albumin (ALB), alkaline phosphatase (AKP), total bile acid (TBA), prealbumin (PA), urea nitrogen (N), creatinine (Cr), PT, international normalized ratio (INR), serum potassium (K), serum sodium (Na), serum chlorine (Cl), hepatitis B surface antigen (HBsAg), e antigen (HBeAg), e antibody (HBeAb), core antibody (HBcAb), HBV DNA, AFP, and blood glucose.

### 2.5 Main reagents and instruments

WBC, NE%, LY%, and EO% were tested using Sysmex XN-9000 (Kobe, Japan); ALT, AST, γ-GT, TBil, ALB, AKP, TBA, K, Na, Cl, and blood glucose were tested using HITACHI7600-120 (Tokyo, Japan); PT was tested using STA COMPACT (France); HBsAg, HBeAg, HBeAb, and HBcAb were tested using CHEMCLIN1500 Automatic chemical immunoanalyzer (Beijing, China); HBVDNA was tested using real-time fluorescence quantitative PRC (ABI7500, Massachusetts, America); AFP was tested using ELISA method (KALANG, Shanghai, China). All specimens were tested at the Clinical Laboratory Center of Shanghai Changhai Hospital.

### 2.6 HBV-ACLF-related predictive scoring system

Currently, maturely and widely used predictive scoring criteria for liver failure include CTP score, MELD, MELD-Na, ALBI score, and CLIF-SOFA score. The CTP score was first proposed by Child and Turcotte in 1964, which included five indicators such as serum bilirubin, albumin, ascites, hepatic encephalopathy, and nutritional status. In 1973, Pugh modified the criterion to form a Child-Pugh scoring model. The model replaced nutritional status with PT and divided each indicator into grades of 1–3 points according to the degree of the condition, where grade A was 5–6 points, grade B was 7–9 points, and grade C was 10–15 points. It was the most classic model for assessing the condition and prognosis of patients with cirrhosis. Since the model factors subjected to influence by therapeutic factors such as diuretics, infusion of albumin, and renal function factors were excluded, there was deficiency in the evaluation of patients with liver failure.

The MELD score was first proposed by Malinchoc. The scoring model could be used to evaluate the need for transjugular portal-body bypass in patients with cirrhosis. In 2001, Kamath et al. revised the formula: *R* = 9.6 × (Cr [mg/dL]) + 3.8 × loge (TBil [mg/dL]) + 11.2 × loge (INR) + 6.4 × cause (cholestasis or alcohol is 0 and others are 1). This model was recommended by the US Organ Distribution Network in 2002 as a standard for liver function assessment and prognosis judgment in patients undergoing liver transplantation surgery, thus enjoying wide application. On this basis, Biggins et al. [9] carried out a prospective multicenter study on 753 patients with end-stage liver disease and established the MELD-Na scoring system, *R* = MELD + 1.59 × (135 − Na [mmol/L]).

Albumin–bilirubin (ALBI) score was a recently developed new model that combines serum albumin and bilirubin to assess the severity of liver dysfunction. The previous study [17] showed that ALBI score was more accurate in predicting long-term survival in patients with HBV-associated cirrhosis than classical scores such as Child-Pugh or MELD. High predictive scores predicted poor prognosis of patients with HBV-ACLF in the short term.

### 2.7 Prognostic scoring system

The prognostic scoring system included MELD score, MELD-Na score, and ALBI score. MELD score formula [14]: 3.78 × LN (serum bilirubin [mg/dL]) + 11.2 × LN (INR) + 9.57 × LN (serum creatinine [mg/dL]) + 6.43 (cause: biliary or alcoholic 0, other 1). MELD-Na formula [9]: MELD + 1.59 × (135 − Na). Patients with serum sodium >135 mmol/L were calculated as 135 mol/L; <120 mmol/L were calculated as 120 mol/L; 120–135 mmol/L calculated as specific values.

### 2.8 Prognostic criteria

The prognostic criteria of patients in this study were based on *Guidelines for the Diagnosis and Treatment of Liver Failure* [16] issued by China in 2012. Good prognosis was required to meet the following conditions: (1) clinical symptoms were significantly improved, and hepatic brain symptoms disappeared. (2) The symptoms of jaundice and ascites were significantly improved. (3) The liver function was significantly improved (total bilirubin < 5ULN; PTA > 40%). Poor prognosis included the following conditions: (1) patients who died from liver failure or related complications during hospitalization (other causes of death were not included). (2) Due to poor effect of clinical treatment and continuous deterioration of the condition, through clinical evaluation, patients who were likely to have severe and life-threatening complications within 1 week and who chose to be automatically discharged from hospital or receive liver transplantation.

### 2.9 Statistical analysis

The data from 220 patients were included in the statistical analysis, including 120 cases in good prognosis group and 100 cases in poor prognosis group.

Continuous variables were tested for normality with the Kolmogorov–Smirnov test. Data are presented as mean ± standard deviation or median (percentile 25 to percentile 75). Categorical variables are expressed as number. When appropriate, continuous variables were compared using Student’s *t*-test or Mann–Whitney *U*-test. Categorical variables were summarized using frequency and percentage and compared using Chi-square test or Fisher’s exact test.

For all possible determinants of poor prognosis, univariate logistic regression analysis was performed. All variables with a *P* value of <0.1 (defined “*a priori*”) were considered relevant and included into the multivariate logistic regression analysis. This second analysis was used to define the factors that were independently associated with poor prognosis. All the analyses were performed using R software. A *P* value of <0.05 was considered to be statistically significant.

Receiver operating characteristic (ROC) curves were constructed for variables that significantly predict poor prognosis. Areas under the curve with the 95% confidence interval (CI) were calculated.

Logistic regression analysis was performed with R 3.5.1 for macOS (R Foundation for Statistical Computing, Vienna, Austria). We have complete patient records in our study without any missing values.

## 3 Results

### 3.1 Inclusion of patients

**Figure 1:**
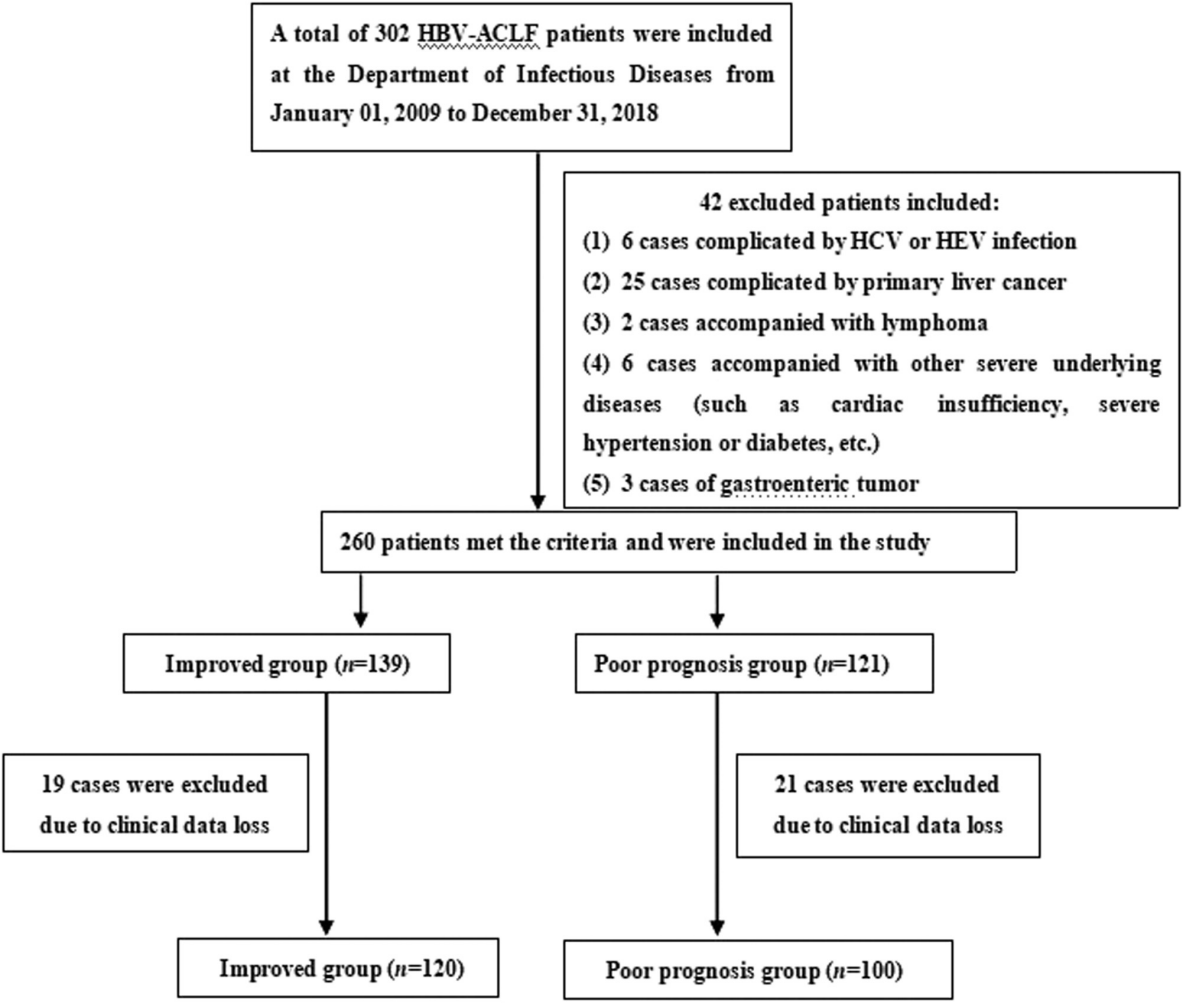
Case screening and registration. There was no statistical difference in the disease stage at admission between the poor prognosis group and the good prognosis group.

A total of 302 HBV-ACLF patients hospitalized at the Department of Infectious Diseases of Changhai Hospital from January 1, 2009, to October 2018 were included in the study. According to the inclusion and exclusion criteria, 42 cases were excluded due to complication of other hepatitis, tumor, or other underlying diseases, and a total of 260 cases were included in the study. Of the 260 patients, 40 cases were not included in the statistics due to clinical data loss. The list of related data was deleted. Of the remaining 220 patients, there were 190 males and 30 females. According to the prognosis of patients, they were divided into good prognosis group and poor prognosis group. There were 120 cases in the good prognosis group, accounting for 54.55%, and 100 cases in the poor prognosis group, accounting for 45.45% (see Figure 1).

### 3.2 Demographic data

There was no statistically significant difference in sex ratio between the two groups, with recovered group versus poor prognosis group (male/female, 100/20 vs 90/10, *p* = 0.1). The age of poor prognosis group was significantly higher than that of recovered group (49.83 ± 11.74 years vs 44.62 ± 12.52 years, *P* = 0.002; see Table 1).

**Table 1:** Demographic information of patients

Variable	Good prognosis group	Poor prognosis group	Statistical value	*P*
Male* (%)	100 (83.3)	90 (90)	1.421	0.16
Age** (year)	44.6 ± 12.5	49.8 ± 11.7	3.0389	<0.01
Hepatic encephalopathy* (yes/no)	7/113	46/54	5.9825	<0.01
Ascites* (yes/no)	52/68	79/21	5.1905	<0.01

^*^Chi-squared test.

^**^ *t*-Test.

### 3.3 Clinical indicators of HBV-ACLF

The proportion of ascites in poor prognosis group was significantly higher than that in recovered group (*P* < 0.001). The incidence of hepatic encephalopathy was also significantly higher than that in recovered group (*P* < 0.001). There was no statistically significant difference in sex ratio between the two groups. Among the two groups of patients with different prognoses, the TBil, Cr, WBC, NEU, and INR levels of poor prognosis group were significantly higher than those of good prognosis group (*P* < 0.05). The ALB, ALT, Na, Cl, RBC, and PLT levels of good prognosis group were significantly higher than those of poor prognosis group (*P* < 0.05). There were no statistically significant differences in AST, AKP, TBA, γ-GT, PA, Glu, or other indicators between the two groups (see Table 2).

**Table 2:** Clinical indicators of patients (baseline)

	Good prognosis group	Poor prognosis group	Statistical value	*P*
Hepatic encephalopathy* (yes/no)	7/113	46/54*	5.9825	<0.01
Ascites* (yes/no)	52/68	79/21*	5.19	<0.01
TBil** (µmmol/L)	259.49 ± 129.42	359.91 ± 180.15**	4.34	<0.01
ALB** (g/L)	32.08 ± 5.16	30.07 ± 5.33**	−2.71	0.01
ALT** (U/L)	477.32 ± 486.84	277.41 ± 340.25**	−3.21	<0.01
AST** (U/L)	334.73 ± 327.21	280.59 ± 314.96**	−1.23	0.21
AKP** (U/L)	147.12 ± 62.47	154.39 ± 64.24**	0.85	0.40
Cr** (µmmol/L)	70.45 ± 18.65	87.83 ± 62.38**	2.51	0.01
TBA** (μmol/L)	197.85 ± 106.81	447.25 ± 2390.65**	0.80	0.30
γ-GT** (U/L)	134.91 ± 128.17	122.91 ± 138.88**	−0.66	0.51
PA** (mg/L)	60.02 ± 38	56 ± 32.88**	−0.83	0.40
Glu** (mmol/L)	4.57 ± 1.65	5.4 ± 4.72**	1.62	0.10
Na** (mmol/L)	134.28 ± 2.24	133.1 ± 3.42**	−2.75	<0.01
Cl** (mmol/L)	100.99 ± 5.89	96.93 ± 5.78**	−4.68	<0.01
WBC** (×10^9^/L)	6.18 ± 2.89	8.29 ± 4.41**	3.85	<0.01
NEU × 10^9^/L** (%)	3.81 ± 2.08	6.3 ± 5.61**	4.72	<0.01
RBC × 10^12^/L**	4.01 ± 0.79	3.71 ± 1**	−2.4	0.02
PLT × 10^9^/L**	128.61 ± 63.41	93.19 ± 47.9**	−4.19	<0.01
INR**	1.73 ± 1.19	2.67 ± 2.08**	4.25	<0.01

^*^Chi-square test.

^**^ *t*-Test.

### 3.4 Establishment of new prediction model

**Figure 2:**
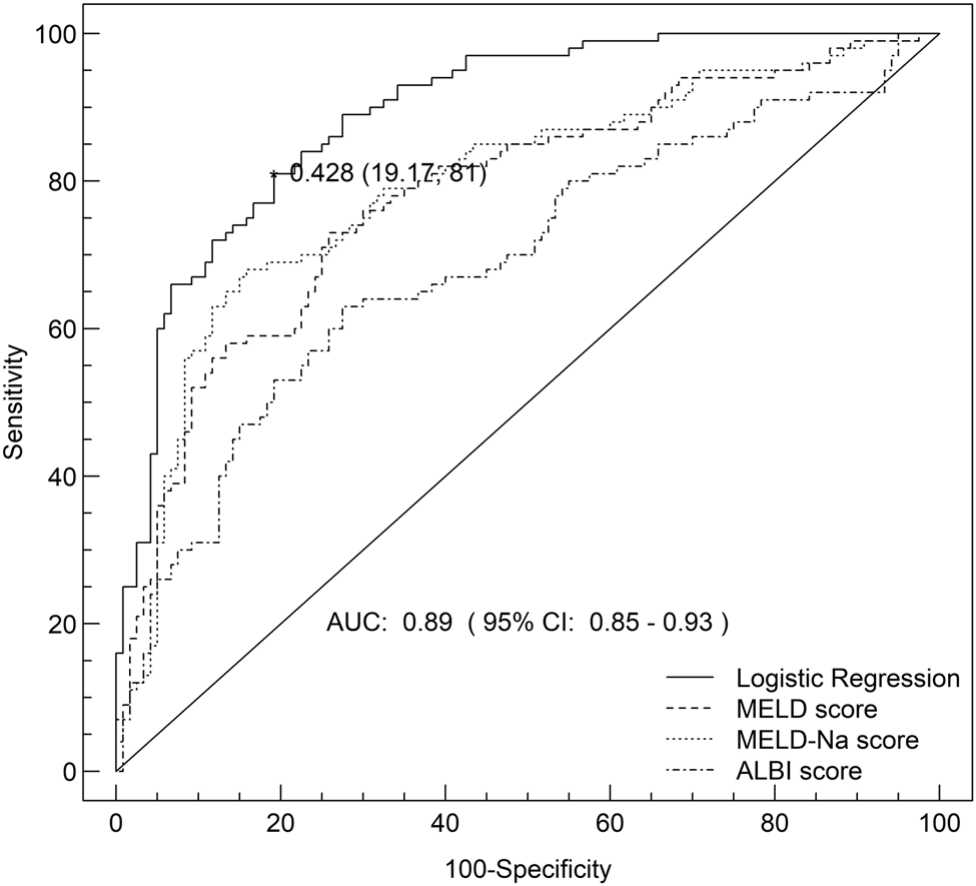
ROC curve corresponding to LR(*p*), MELD, MELD-Na, and ALBI. LR(*p*) was the newly established prediction model in this study.

In this study, demographic information, baseline clinical indicators, as well as other potential parameters that might affect prognosis in the two groups were subjected to analysis. Many potential parameters for poor prognosis in HBV-ACLF patients were defined, including advanced age, ascites, hepatic encephalopathy, high TBil, Cr, WBC, NEU, and INR levels and low ALB, ALT, Na, Cl, RBC, and PLT levels. Univariate and multivariate logistic regression analyses were used to determine independent risk factors for poor prognosis (see Table 3). The ROC curve was constructed and compared with other prediction models. The results showed that the specificity, sensitivity, and area under the ROC curve of the new model were significantly higher than those of the other three models, and had better predictive value (see Figure 2). The newly established model is as follows: Logistic regression (*p*) = 1/(1 + *e* ^−*Z*^), where *z* = 10.0127 + 0.3687 × NEUT (%) − 0.0082 × PLT + 1.8157 × hepatic encephalopathy. The area under the ROC curve was 0.89, 95% CI: 0.85–0.93, specificity was 80.83%, and sensitivity was 81%.

**Table 3:** Univariate and multivariate logistic regression analysis

Variable	Univariate analysis		Multivariate analysis	
	OR (95% Cl)	*P* value	OR (95% Cl)	*P* value
Sex	1.8 (0.817–4.201)	0.16		
Age	1.0359 (1.013–1.06)	<0.01		
Hepatic encephalopathy	13.7513 (6.171–35.167)	<0.01	6.1455 (2.362–18.022)	<0.01
Ascites	4.9194 (2.733–9.135)	<0.01		
TBiL	1.0042 (1.002–1.006)	<0.01		
ALB	0.9267 (0.875–0.977)	0.01		
ALT	0.9988 (0.998–0.999)	0.01		
AST	0.9995 (0.999–1)	0.22		
AKP	1.0018 (0.998–1.006)	0.40		
Creatinine	1.0128 (1.004–1.024)	0.01		
TBA	1.0011 (1–1.004)	0.42		
γ-gt	0.9993 (0.997–1.001)	0.51		
PA mg/L	0.9967 (0.989–1.004)	0.41		
Blood glucose	1.0948 (0.997–1.237)	0.10		
Sodium	0.8518 (0.751–0.946)	0.01		
Chlorine	0.8693 (0.818–0.919)	<0.01		
WBC	1.1883 (1.093–1.303)	<0.01		
NEUT (%)	1.3454 (1.197–1.533)	<0.01	1.4458 (1.108–1.862)	<0.01
RBC	0.6892 (0.505–0.93)	0.02		
PLT	0.9882 (0.982–0.993)	<0.01	0.9918 (0.983–1)	0.04
INR	2.3978 (1.641–3.677)	<0.01		

## 4 Discussion

As we all know, HBV-ACLF, a clinically serious syndrome, has numerous occurrence and development factors. The results of this study showed and confirmed some factors affecting its prognosis.

The age factor was significantly associated with HBV-ACLF. This study found that the age of poor prognosis group was significantly higher than that of good prognosis group. Therefore, age might be a potential parameter influencing the prognosis of HBV-ACLF. In this study, white blood cell level and neutrophil ratio were positively correlated with the patients’ mortality. The white blood cell and neutrophil levels of poor prognosis group were significantly higher than those of good prognosis group. Elevation of white blood cell and neutrophil levels suggested an aggravation of infection or inflammation. The previous study [18] suggested that aggravation of infection and inflammatory response and imbalance of inflammatory factors in the body were important factors affecting the prognosis of ACLF. This conclusion indicated that white blood cell and neutrophil levels were also important assessment indicators affecting the prognosis of patients with ACLF. In addition, the red blood cell and platelet levels of poor prognosis group were significantly lower than those of good prognosis. A decrease in red blood cell and platelet levels was associated with the severity of cirrhosis and the resulting hypersplenism. It could be inferred that patients in poor prognosis group had worse basis of chronic liver disease and were more prone to serious complications.

In terms of liver function, patients with liver failure may develop ascites due to the decreased ability of the liver synthesizing protein, low colloid osmotic pressure, portal hypertension, aldosteronism, increase in antidiuretic hormone, insufficient effective circulating blood volume, and many other factors. In addition, hepatic encephalopathy is a serious complication of end-stage liver disease, which plays a promoting role on the prognosis of ACLF. In this study, the incidence of ascites and hepatic encephalopathy in the poor prognosis group was significantly higher than that of good prognosis; therefore, the occurrence of ascites and hepatic encephalopathy suggested a poor prognosis of ACLF patients.

Refractory hyponatremia and persistent damage of the renal function are common clinical manifestations in ACLF patients. Especially in the late stage of ACLF, hyponatremia is irreformable, and the continuous increase in urea nitrogen and creatinine levels is still easy to cause hepatorenal syndrome. In this study, the creatinine level of poor prognosis group was significantly higher than that of good prognosis group, while the blood sodium level was significantly lower than that of good prognosis group. Biggins et al. included serum sodium in the MELD score and improved it, confirming the importance of changes in the blood sodium level in predicting the prognosis of ACLF patients. Therefore, monitoring of blood sodium and serum creatinine levels should be strengthened during clinical diagnosis and treatment.

ACLF patients suffer from severe liver damage, massive destruction of liver cells, reduction in liver transaminase synthesis, decrease in blood coagulation factors, and decline in bilirubin metabolism due to various factors, which leads to decrease in the serum transaminase level, elevation of the bilirubin level, and coagulation disorders. The liver function and coagulation function in both groups were significantly abnormal, but the total bilirubin level and INR level of poor prognosis group were significantly higher than those of good prognosis group, while the ALT level was significantly lower than that of good prognosis group.

The HBV-ACLF scoring system can help clinicians to predict the early prognosis of the disease, assess the severity of the disease, and provide guidance for the selection of active and effective treatments. However, due to different factors such as genetic polymorphism, race, and disease occurrence and development, each scoring system has a certain degree of defects. This study assessed HBV-ACLF patients with clinically common scoring systems such as MELD, MELD-Na, and ALBI. For example, ascites could be affected by diuretics, and albumin level could be changed by intravenous infusion. In addition, the renal function was not reflected in the model, which led to the CTP score not fully reflecting the severity and prognosis of the disease. Therefore, the CTP score was not included in the study. The results showed that MELD, MELD-Na, and ALBI scores had also good predictive value for ACLF patients.

This study determined the related potential factors affecting the prognosis of ACLF and established a new prediction model through univariate and multivariate logistic regression analysis: LR(*p*) = 1/(1 + *e* ^−*z*^), where *z* = 10.0127 + 0.3687 × NEUT (%) − 0.0082 × PLT + 1.8157 × hepatic encephalopathy. The AUC was 0.89 (95% CI: 0.85–0.93) for logistic regression, 0.78 (95% CI: 0.72–0.84) for MELD, 0.79 (95% CI: 0.73–0.86) for MELD Na, and 0.69 for ALBI scores (95% CI: 0.62–0.76). Delong’s test was used to compare the ROC curves. The AUC of our logistic regression was significantly higher than those of MELD (*p* < 0.01), MELD Na (*p* < 0.01), and ALBI (*p* < 0.01).

Hepatic encephalopathy, NEU (%), and PLT level are independent risk factors for predicting the prognosis of HBV-ACLF. Hepatic encephalopathy is also one of the serious complications of ACLF. In addition, as for individual indicators, the neutrophil ratio and platelet level are independent predictive factors for the prognosis of HBV-ACLF and both have a high predictive value. The neutrophil ratio indicates the severity of the infection or inflammatory response, while platelets reflect the coagulation function of the patients. The aforementioned aspects are of great significance in the occurrence, development, and clinical diagnosis and treatment of ACLF. The number of samples included in this study was derived from a single center, which may have an impact on the accuracy of the scoring system, and multicenter large sample size would be required for further validation of the prediction system in the future. In addition, this study was a retrospective study. All the enrolled patients were Asians of yellow race, so the race was relatively limited.

The mechanism of occurrence and development of ACLF is complicated, and it is easily interfered by various factors during the progression of the disease, and the disease can involve in multiple organs, thereby affecting its prognosis. In addition to traditional assessment indicators, many new indicators and new prediction models are emerging, providing clinicians with a variety of lessons. Individual indicators or individual models are still unable to fully assess the prognosis of the disease. In future, to more accurately judge the prognosis of ACLF, on one hand, is necessary to explore new indicators with higher sensitivity and specificity; on the other hand, it is necessary to carry out statistics on large-scale clinical data and to formulate more reasonable prediction models.

## References

[1] Liver Failure and Artificial Liver Group of Infectious Diseases Branch of Chinese Medical Association, Severe Hepatitis and Artificial Liver Group of Hepatology Association of Chinese Medical Association. Guidelines for diagnosis and treatment of liver failure (2012). Chin J Hepatol. 2013;21(3):177–83.

[2] Moreau R, Jalan R, Gines P, Pavesi M, Angeli P, Cordoba J, et al. Acute-on-chronic liver failure is a distinct syndrome that develops in patients with acute decompensation of cirrhosis. Gastroenterology. 2013;144(7):1426–37. 10.1053/j.gastro.2013.02.042 23474284

[3] Garg H, Kumar A, Garg V, Sharma BC, Sarin SK. Clinical profile and predictors of mortality in patients of acute-on-chronic liver failure. Dig Liver Dis. 2012;44(2):166–71. 10.1016/j.dld.2011.08.029 21978580

[4] Jalan R, Gines P, Olson JC, Mookerjee RP, Moreau R, Garcia-Tsao G, et al. Acute-on chronic liver failure. J Hepatol. 2012;57(6):1336–48. 10.1016/j.jhep.2012.06.026 22750750

[5] Vaid A, Chweich H, Balk EM, Jaber BL. Molecular adsorbent recirculating system as artificial support therapy for liver failure: a meta-analysis. ASAIO J. 2012;58(1):51–9. doi: 10.1097/MAT.0b013e31823fd077. 22210651

[6] Shen Y, Wang XL, Wang B, Shao JG, Liu YM, Qin Y, et al. Survival benefits with artificial liver support system for acute-on-chronic liver failure: a time series-based meta-analysis. Medicine. 2016;95(3):e2506. doi: 10.1097/MD.0000000000002506. PMC4998263 26817889

[7] Chen JJ, Huang JR, Yang Q, Xu XW, Liu XL, Hao SR, et al. Plasma exchange-centered artificial liver support system in hepatitis B virus-related acute-on-chronic liver failure: a nationwide prospective multicenter study in China. Hepatobiliary Pancreat Dis Int. 2016;15(3):275–81. doi: 10.1016/S1499-3872(16)60084-X. 27298103

[8] Durand F, Valla D. Assessment of the prognosis of cirrhosis: Child-Pugh versus MELD. J Hepatol. 2005;42(Suppl):S100–7, [PMID: 15777564. doi: 10.1016/j.jhep.2004.11.015]. 15777564

[9] Biggins SW, Kim WR, Terrault NA, Saab S, Balan V, Schiano T, et al. Evidence-based incorporation of serum sodium concentration into MELD. Gastroenterology. 2006;130(6):1652–60. 10.1053/j.gastro.2006.02.010 16697729

[10] Gao F, Sun L, Ye X, Liu Y, Liu H, Geng M, et al. Development and validation of a prognostic model for acute-on-chronic hepatitis B liver failure. Eur J Gastroenterol Hepatol. 2017;29(6):669–78. 10.1097/MEG.0000000000000854 28195876

[11] Wu T, Li J, Shao L, Xin JJ, Jiang LY, Zhou Q, et al. Development of diagnostic criteria and a prognostic score for hepatitis B virus-related acute-on-chronic liver failure. Gut. 2018;67(12):2181–91. 10.1136/gutjnl-2017-314641 28928275

[12] Wang GQ, Wang FS, Chen J, et al. Guidelines for the prevention and treatment of chronic hepatitis B (2015). J Clin Hepatol. 2015;31(12):1941–60.

[13] Liver Failure and Artificial Liver Group of Infectious Diseases Branch of Chinese Medical Association, Severe Hepatitis and Artificial Liver Group of Hepatology Association of Chinese Medical Association. Guidelines for diagnosis and treatment of liver failure (2012). J Pract Hepatol. 2013;16(3):210–6.

[14] Kamath PS, Wiesner RH, Malinchoc M, Kremers W, Therneau TM, Kosberg CL, et al. A model to predict survival in patients with end-stage liver disease. Hepatology. 2001;33:464–70, [PMID: 11172350. doi: 10.1053/jhep.2001.22172]. 11172350

[15] Johnson PJ, Berhane S, Kagebayashi C, S Satomura, Teng M, Reeves HL, et al. Assessment of liver function in patients with hepatocellular carcinoma: a new evidence-based approach – the ALBI grade. J Clin Oncol. 2015;33(6):550–8. 10.1200/JCO.2014.57.9151 PMC4322258 25512453

[16] Li LJ, Duan ZP. Guidelines for diagnosis and treatment of liver failure (2012). Organ Transplant. 2013;21(4):210–6.

[17] Chen B, Lin S. Albumin-bilirubin (ALBI) score at admission predicts possible outcomes in patients with acute-on-chronic liver failure. Medicine. 2017;96(24):e7142. 10.1097/MD.0000000000007142 PMC5478326 28614241

[18] Zou Z, Li B, Xu D, Zhang Z, Zhao JM, Zhou G, et al. Imbalanced intrahepatic cytokine expression of interferongamma, tumor necrosis factor-alpha, and interleukin-10 in patients with acute-on chronic liver failure associated with hepatitis B virus infection. J Clin Gastroenterol. 2009;43:182–90. 10.1097/MCG.0b013e3181624464 18633332

